# Correction for: Circular RNA circRGNEF promotes bladder cancer progression via miR-548/KIF2C axis regulation

**DOI:** 10.18632/aging.204791

**Published:** 2023-05-31

**Authors:** Chen Yang, Qiong Li, Xinan Chen, Zheyu Zhang, Zezhong Mou, Fangdie Ye, Shengming Jin, Xiang Jun, Feng Tang, Haowen Jiang

**Affiliations:** 1 Department of Urology, Huashan Hospital, Fudan University, Shanghai 200040, China; 2 Fudan Institute of Urology, Huashan Hospital, Fudan University, Shanghai 200040, China; 3 National Clinical Research Center for Aging and Medicine, Fudan University, Shanghai 200032, China; 4 Department of Pathology, Huashan Hospital, Fudan University, Shanghai 200040, China; 5 Shanghai Cancer Center, Fudan University, Shanghai 200040, China; 6 Department of Urinary Surgery, Tongji Hospital, Tongji University School of Medicine, Shanghai 200065, China

**Keywords:** circular RNA, circRGNEF, miR-548, bladder cancer, KIF2C

**This article has been corrected:** The authors found that in **Figure 6J** the wrong images were used to illustrate migration of UM-UC-3 cells treated with si-circRGNEF+inhibitor or si-circRGNEF+KIF2C. The same error occurred in **Figure 7I**, where migration images of UM-UC-3 cells treated with negative control (NC) or T24 cells treated with mimic+KIF2C were incorrect. The authors corrected these errors with the images from the original sets of experiments, the same images used for the statistical analysis. The authors also found that the bar graph in **Figure 7M**, which should have represented statistical analysis data for UM-UC-3 cell invasion, was mistakenly replaced with duplicate UM-UC-3 migration data. The authors replaced the **Figure 7M** bar graph with the correct graph using the original data. These corrections do not change the content of the publication and do not affect the conclusions drawn from this research.

Corrected **Figures 6 and 7** are presented below.

**Figure 6:**
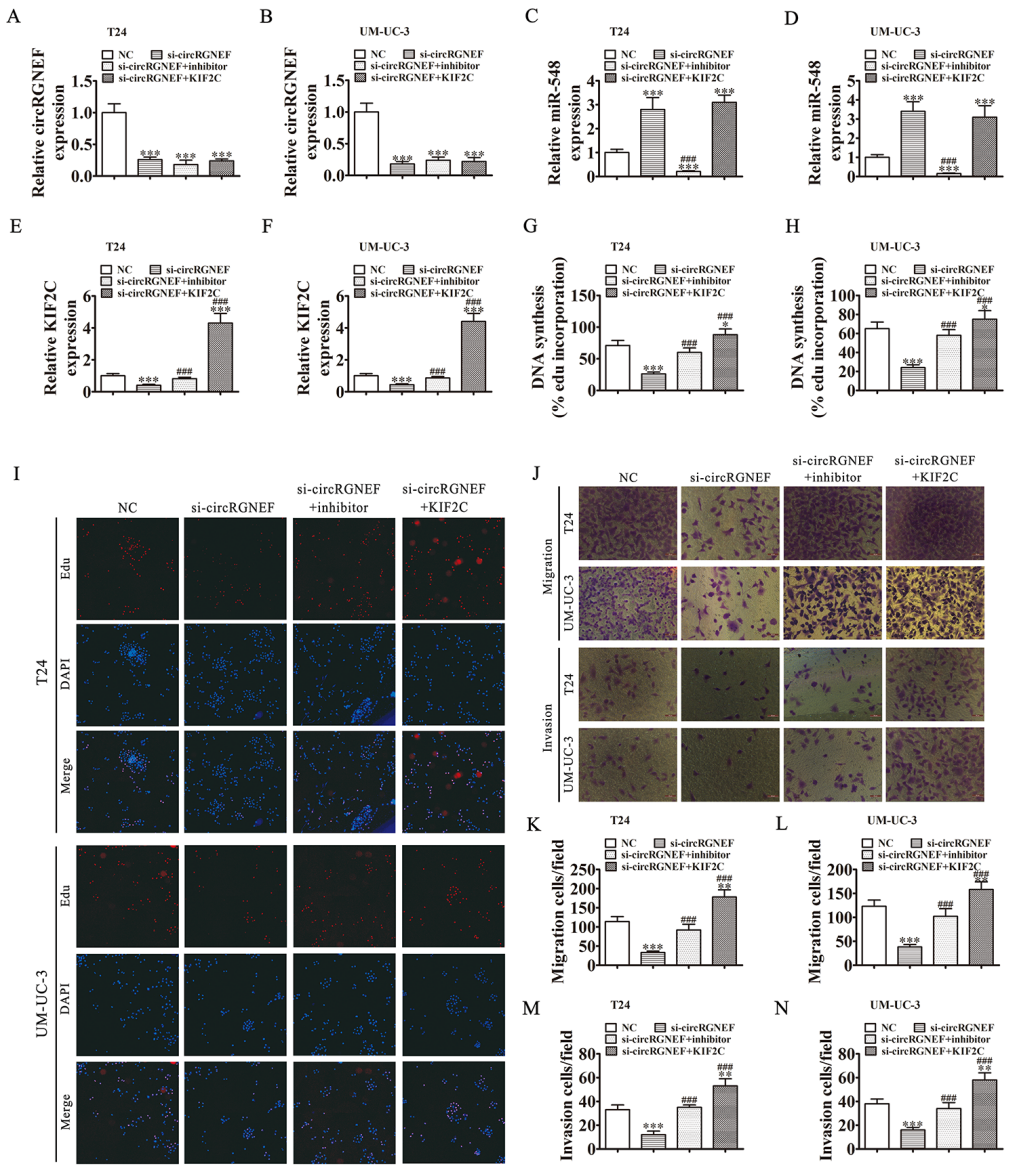
**Downregulation of miR-548 or overexpression of KIF2C restored proliferation, migration, and invasion after circRGNEF silencing.** (**A**–**F**) RT-qPCR shows the expression of circRGNEF (**A**, **B**), miR-548 (**C**, **D**), and KIF2C (**E**, **F**) in T24 and UM-UC-3 cells following transfection or treatment with NC, si-circRGNEF, miR-548 inhibitor, KIF2C overexpression vector (KIF2C) single or combined. Data are presented as the mean ± SD. ***P < 0.001 vs. NC. ^###^*P* < 0.001 vs. si-circRGNEF. (**G**–**I**) Cell proliferation was analyzed by EdU assays. Data are presented as the mean ± SD. ***P < 0.001 vs. NC. ^###^*P* < 0.001 vs. si-circRGNEF. (**J**–**N**) Cell migration and invasion were assessed in T24 and UM-UC-3 cells using Transwell assays. Data are presented as the mean ± SD. ***P < 0.001 vs. NC. ^###^*P* < 0.001 vs. si-circRGNEF.

**Figure 7:**
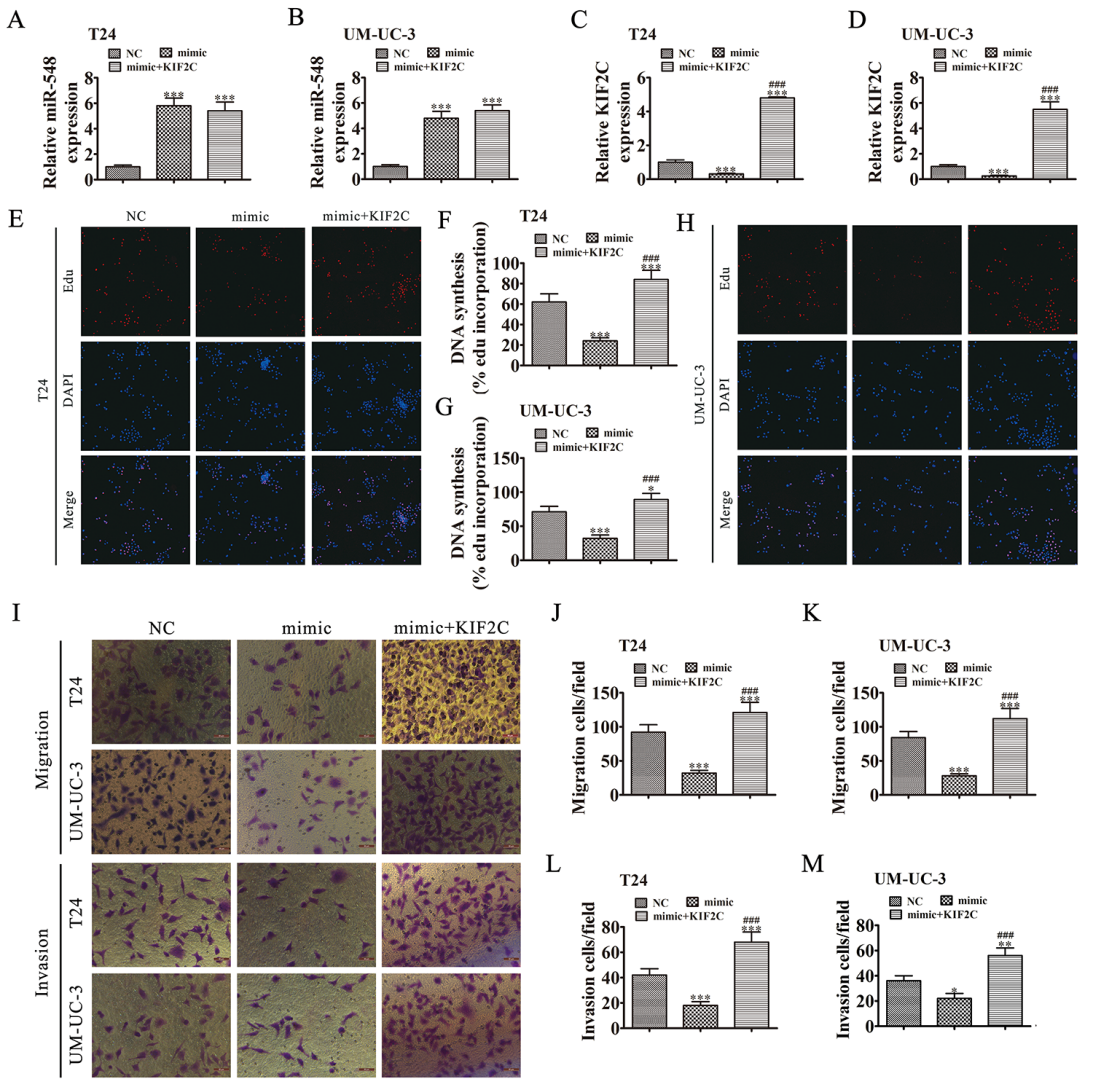
**KIF2C overexpression reversed miR-548-induced cell migration, invasion, and growth inhibition *in vitro*.** (**A**–**D**) T24 and UM-UC-3 cells were transfected with miR-548 mimics with or without the KIF2C overexpression vector. RT-qPCR shows the expression of miR-548 (**A**, **B**) and KIF2C (**C**, **D**) in T24 and UM-UC-3 cells. Data are denoted by the mean ± SD. ***P < 0.001 vs. NC. ^###^*P* < 0.001 vs. mimic.  (**E**–**H**) EdU assay showing the proliferation of T24 (**E**, **F**) and UM-UC-3 (**G**, **H**) cells. Data are denoted by the mean ± SD. *P < 0.05, ***P < 0.001. ^###^*P* < 0.001 vs. mimic. (**I**–**M**) Cell migration and invasion were determined in T24 and UM-UC-3 cells by Transwell assays. Data are denoted by the mean ± SD. ***P < 0.001 vs. NC. ^###^*P* < 0.001 vs. mimic.

